# Pathological proliferation: a potential mechanism for poor CD4^+^ T cell recovery in people living with HIV

**DOI:** 10.3389/fcimb.2024.1344778

**Published:** 2024-03-27

**Authors:** Yang Zhang, Jiahao Ji, Kaidi Xie, Miaotian Cai, Rui Wang, Xin Zhang, Xue Chen, Yulin Zhang, Hao Wu, Wen Wang, Zhen Li, Tong Zhang

**Affiliations:** ^1^ Center for Infectious Diseases, Beijing Youan Hospital, Capital Medical University, Beijing, China; ^2^ Beijing Institute of Sexually Transmitted Disease Prevention and Control, Beijing, China; ^3^ Department of Respiratory and Critical Care Medicine, Beijing Youan Hospital, Capital Medical University, Beijing, China; ^4^ Beijing Key Laboratory of HIV/AIDS Research, Beijing Youan Hospital, Beijing, China

**Keywords:** pathological proliferation, immunological non-responder (INR), CD4+ T cells, human immunodeficiency virus - HIV, mass cytometry

## Abstract

**Background:**

People living with HIV (PLWH) fail to achieve normalization of CD4^+^ T cell counts and function, especially in immunological non-responders (INRs). The frequencies of Ki67^+^CD4^+^ T cells were inversely associated with CD4^+^ T cell counts in HIV infected patients. Early ART did not normalize CD4^+^ T cell proliferation. However, the features of the abnormal proliferation CD4^+^ T cell in INRs are far from known.

**Method:**

PLWH were divided into INRs (n= 16) and immunological responders (IRs, n= 53) groups. Mass cytometry was applied to peripheral blood T cells to profile the immune cells and liquid chip technique was used to measure plasma levels of cytokines and chemokines. Correlation analyses were conducted to evaluate associations between the degree of CD4^+^ T cell proliferation and immune function.

**Results:**

The percentage of Ki67^+^ CD4^+^ T cells were significant higher in INRs, and we defined these cells with significant higher level of Ki67, as over-proliferating cells. No significant difference of markers’ expression (HLA-DR, CD38, CD57, PD-1, PD-L1, CD107a, perforin) was found between INRs and IRs. Compared with naïve CD4^+^ T cells in INRs, Ki67^+^ CD4^+^ T cells exhibited lower levels of CD57 and CD38. Whereas Ki67^+^ T cells exhibited higher levels of CD38 and CD57 and activation compared with differentiated mature central memory CD4^+^ T cells and effector memory CD4^+^ T cells. Ki67^+^ cells did not show higher levels of senescence and activation compared to certain Ki67^-^ CD4^+^ central memory T cells in IRs. Furthermore, Ki67^+^ CD4^+^ Tcm cells exhibited positive correlations with pro-inflammatory cytokines.

**Conclusion:**

We proposed and validated the hypothesis of “pathological proliferation” in INRs: excessive proliferation of CD4^+^ T cells in INRs may be accompanied by aberrant activation, senescence and loss of immune function. Eventually, such over-proliferating but poor-quality cells in INRs result in incomplete recovery of both CD4^+^ T cell counts and function. An intervention that enhancing the proliferative capacity or functional ability or both of CD4^+^ T cell in INRs might therefore be beneficial.

## 1 Introduction

The development and roll out of antiretroviral therapy (ART) has led to a reversal of the devastating mortality of acquired immunodeficiency syndrome (AIDS) (Bekker, 2023). Despite substantial progress, the epidemic is far from over and remains a major global health problem (Guilamo-Ramos et al., 2023). People living with HIV (PLWH) fail to achieve normalization of CD4^+^ T cell counts and function despite persistent blood virological suppression, especially in immunological non-responders (INRs, usually defined as PLWH under ART with viremia and CD4^+^ T cell counts < 350/μl in the blood) (Rb-Silva et al., 2019; Bono et al., 2022). This severe immunological dysfunction is thought to contribute not only to HIV disease progression, but also to mortality and emerging non-AIDS morbidity (Fauci and Lane, 2020). To date, there is still a lack of effective adjunct medical therapy to enhance CD4^+^ T cell counts and function for PLWH, especially INRs (Zhang et al., 2021).

Saidakova et al. demonstrated that the frequencies of Ki67^+^CD4^+^ T cells were inversely associated with CD4^+^ T cell counts in HIV infected patients, and INRs have higher frequencies of Ki67^+^PD-1^+^CD4^+^ T cells (Saidakova et al., 2018). Our previous research has shown that persistent CD4^+^ T cell proliferation precede incomplete CD4^+^ T cell recovery in people with acute HIV infection with early ART (Li et al., 2023). Early ART normalized CD4^+^ T cell activation but not their proliferation. CD4^+^ T cell proliferation, enriched in PD-1^+^ T cells, was persisted and negatively associated with CD4^+^ T cell counts after ART (Li et al., 2023). Therefore, we proposed the hypothesis of “pathological proliferation” in INRs: there is excessive proliferation of CD4^+^ T cell in INRs, and this overproliferation of CD4^+^ T cell dies excessively at the same time, resulting in a decrease in the final CD4^+^ T cell counts.

Moreover, this over-proliferate of CD4^+^ T cell may also be accompanied by abnormal activation and loss of immune function. A heterogeneous population of dysfunctional T cells was identified in the immune microenvironment of tumors, which expressed PD-1, had the highest clone expansion (Li et al., 2019), and were highly proliferated and dynamically differentiated (Guo et al., 2018). However, the features of the heterogeneous population of dysfunctional CD4^+^ T cell (pathological proliferation) in INRs are far from known.

In this study, in order to test the hypothesis of “pathological proliferation”, we measured CD4^+^ T cell counts and function, as well as common cytokine levels, in peripheral blood of INRs and immunological responders (IRs). By describing the main characteristics of “pathological proliferation”, it is hoped to increase the understanding of poor immune reconstitution and to find potential targets to INRs.

## 2 Materials and methods

This study has been approved by the Ethics Committee of Beijing Youan Hospital, Capital Medical University. Each participant has been informed about the purpose of the study, and a written informed consent was obtained. For experiments involving humans, the research was conducted in accordance with the Declaration of Helsinki of the World Medical Association revised in 2013.

### 2.1 Participants

A total of 69 individuals with HIV infection were enrolled at Beijing Youan Hospital, Capital Medical University, from April 1, 2022, to September 31, 2022. Patients presenting with opportunistic infection, autoimmune diseases, epilepsy, traumatic brain injury, substance use, undergoing suppressive therapy, or other severe disease were excluded from the study. All the patients treated with ART with undetectable serum viral loads. The clinical characteristics of the subjects (including demographics, CD4^+^ T cell counts, HIV RNA load, date of HIV infection, duration of HIV infection, date of ART initiation, and duration of ART treatment) were obtained from the medical records of the AIDS research cohort at Beijing Youan Hospital. For those with total CD4^+^ T cell counts < 350 cells/µl and/or increase in the CD4^+^ T cell counts < 30% from baseline at 2 years after ART initiation, with an undetectable plasma HIV RNA load were classified into INRs, while individuals with total CD4^+^ T cell counts > 500 cells/µl and/or increase in the CD4^+^ T cell counts > 30% from baseline at 2 years after ART initiation, with an undetectable plasma HIV RNA load were classified into IRs. For each participant, plasma and peripheral blood mononuclear cell (PBMC) samples were stored at −80°C and in liquid nitrogen tanks, respectively. All participants provided written informed consent, as approved by the Beijing Youan Hospital ethics committee (2023/057).

### 2.2 Mass cytometry and data analysis

Heavy metal isotope-tagged monoclonal antibodies are listed in **Supplementary Table 1**. A total of twenty-three custom-designed antibodies were utilized to differentiate a diverse range of immune cells. These antibodies were acquired in a pre-conjugated state from Fluidigm (South San Francisco, United States). The cell labeling procedure adhered to previously established protocols (Hallisey et al., 2023). To summarize, the isolated PBMCs were washed and treated with Cisplatin-195Pt (Fluidigm, 201064) to eliminate deceased cells. Human TruStain FcX was employed for Fc-receptor blocking prior to antibody staining. All antibodies were utilized in accordance with the manufacturer’s instructions. Subsequently, cell samples underwent a washing process and were stained with cell surface antibodies for a duration of 30 minutes while being kept at a low temperature. Following this, the samples labeled with antibodies were washed and subjected to incubation in 125nM Cell-ID Intercalator-Ir (Fluidigm, United States), which was diluted in phosphate-buffered saline (PBS, SigmaAldrich, United States), and stored at a temperature of 4°C. The samples were resuspended at a concentration of 5.5 × 10^5^ cells/mL in double-distilled water containing a final concentration of 10% EQ Beads (Fluidigm, South San Francisco, United States). The samples were subsequently analyzed using the CyTOF2 mass cytometry system (Fluidigm, South San Francisco, United States).

The raw data of each sample were de-barcoded using unique mass tagged barcodes in a double-filtering scheme. A bead normalization method was employed to normalize the.fcs file obtained from various batches. The data underwent meticulous gating using FlowJo 10.8.1 software to eliminate debris and dead cells. Subsequently, CD45^+^CD3^+^ cells were manually gated (**Supplementary Figure 1**) for further R language analysis. The cells were divided into different clusters using PhenoGragh clustering algorithm based on their surface marker expression levels. The t-distributed stochastic neighbor embedding (t-SNE), a visual dimensionality reduction algorithm, was applied for dimensionality reduction and visualization of the high-dimensional data. The distribution of each cluster and marker expression, and difference analysis among each group or different sample types were performed in R software (version 3.6.0).

### 2.3 Cytokine and chemokine assay

A comprehensive cytokine/chemokine assay was performed on plasma samples from different groups using the EMD Millipore’s MILLIPLEX® MAP Human Cytokine/Chemokine/Growth Factor Panel A MAGNETIC BEAD PANEL 96-Well Plate Assay, which utilizes the advanced Luminex® xMAP® technology. Capture antibody-coupled magnetic beads were utilized to selectively bind the target analytes in the plasma. The plate was then wrapped with foil and incubated with gentle agitation on a plate shaker overnight, maintaining a controlled temperature of 2–8 ◦C. Following the incubation period, the plate was washed three times. Subsequently, the plate was incubated with detection reagents, employing gentle agitation on a plate shaker, for a duration of 1 hour at room temperature (24 ◦C). Finally, the advanced Luminex® 200™, HTS, FLEXMAP 3D® software was employed to run the plate and analyze the resulting data.

### 2.4 Statistical analyses

The statistical analysis was conducted using SPSS (version 27.0) and R (version 3.6.0). χ2test or Fisher’s exact test were employed to compare INRs and IRs for categorical variables, while Student’s t-test or Mann-Whitney U-test were used for continuous variables. Spearman correlations were used to investigate the correlation between Ki67 expression and immune function. The results were performed using GraphPad Prism (version 9.5.1) for Windows and R (version 3.6.0). A p value < 0.05 was considered statistically significant.

## 3 Results

### 3.1 Characteristics of the participants

INRs (n = 16) and IRs (n = 53) were enrolled, and the clinical information was described in **Table 1**. The age, time of starting initial ART, duration of HIV infection, and duration of ART of INRs and IRs were matched. Initial CD4^+^ T cell counts, initial CD8^+^ T cell counts, recent CD4^+^ T cell counts, and recent CD8^+^ T cell counts were significant lower in INRs, while initial HIV load was higher in INRs.

**Table 1:** Demographic and clinical characteristics of all subject.

Demographic and clinical data	IRs (n=53)	INRs (n=16)	*P* value
Age, years, mean (SD)	37.05 (7.61)	38.69 (11.65)	>0.05 ^a^
BMI, kg/m2, mean (SD)	22.86 (2.81)	20.96 (1.83)	0.013* ^a^
Initial CD4 count, count/mm3, median (IQR)	409.58 (329.50, 527.72)	112.50 (30.25, 216.50)	<0.001*
Initial CD8 count, count/mm3, mean (SD)	1114.84 (398.28)	827.87 (252.62)	0.008** ^a^
Initial HIV load (copies/mL), median (IQR)	7418.00 (4257.00, 16272.00)	74120.00 (12257.00, 176235.00)	0.007** ^b^
CD4 count at initiation of ART, count/mm3, median (IQR)	387.48 (333.50, 526.92)	143.15 (31.50, 221.00)	<0.001*** ^b^
CD8 count at initiation of ART, count/mm3, median (IQR)	1076.55 (865.23, 1344.36)	874.50 (618.21, 1187.31)	>0.05 ^b^
HIV load at initiation of ART, copies/mL, median (IQR)	7515.00 (4181.25, 15705.00)	66249.00 (3254.00, 135874.00)	>0.05 ^b^
Recent CD4 count, count/mm3, median (IQR)	709.50 (574.25, 931.00)	258.00 (179.00, 325.50)	<0.001*** ^b^
Recent CD8 count, count/mm3, median (IQR)	951.00 (712.50, 1239.81)	560.00 (374.00, 1067.68)	0.046* ^b^
Time to initiate ART after HIV discovery, months, median (IQR)	0.60 (0.40, 7.58)	0.6 (0.28, 2.23)	>0.05 ^b^
Duration of ART, months, median (IQR)	82.35 (55.45, 102.57)	71.50 (46.98, 88.43)	>0.05 ^b^
Duration of HIV infection, months, mean (SD)	88.40 (58.05, 113.10)	79.45 (51.93, 98.95)	>0.05 ^b^

IRs, immunological responders; INRs, immunological non-responders; BMI, body mass index; ART, antiretroviral therapy; *: P < 0.05; **: P<0.01; ***: P<0.001; ^a^ T-test; ^b^ Mann-Whitney U test; IQR, Interquartile range; SD, Standard deviation. The Shapiro-Wilk test was performed to assess the normal distribution of variables. Standard deviation. A statistical significance with two-sided p<0.05 was adopted.

### 3.2 INRs exhibit a greater proportion of proliferating CD4 subpopulation cells

**Figure 1:**
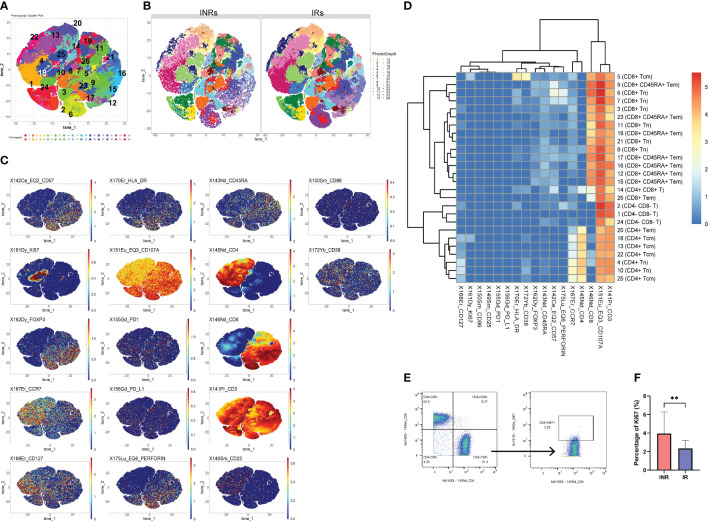
Higher proportion of Ki67^+^ CD4^+^ T cells to total CD4 counts in INRs. **(A)** t-SNE plots of CD3^+^T cells in all participants and the serial numbers of each CD3^+^ T cells cluster. **(B)** t-SNE plots of CD3^+^T cells in INRs and IRs. **(C)** Expression distribution of selected markers across clusters (dark red color, high expression; dark blue color, no expression). **(D)** Heatmap of markers expression for 26 CD3^+^T cell clusters. **(E)** Gating strategy for Ki67^+^ CD4^+^ T cells. **(F)** Comparison of the proportion of CD4^+^ Ki67^+^ T cells between INRs and IRs. IRs, immunological responders; INRs, immunological non-responders; BMI, body mass index Mann–Whitney U-test was used in **(F)**. Data are shown as media and Interquartile range. **p<0.01. Tn, naïve T cells; Tcm, central memory T cells; Tem, effector memory T cells.

We conducted mass cytometry to PBMCs samples from all participants. 9 subtypes of CD3^+^ T cells were analyzed and categorized into 26 clusters based on the expression of markers (**Figure 1A**). The distribution of each cluster in IRs and INRs and expression of markers were depicted in (**Figure 1B**). Our observation in **Figure 1C** revealed a predominant concentration of Ki67 expression in C18 (CD4^+^ T cells), prompting us to direct our attention towards CD4^+^ T cells. Among the 26 clusters of CD3^+^ T cells, C4 and C10 represented CD4^+^ naïve T cells (Tn) (CD3^+^ CD4^+^ CD8^-^ CD45RA^+^ CCR7^+^); C13, C18, C22, C25 represented CD4^+^central memory T cells (Tcm) (CD3^+^ CD4^+^ CD8^-^ CD45RA^-^ CCR7^+^); C20 represented CD4^+^ effector memory T cells (Tem) (CD3^+^ CD4^+^ CD8^-^ CD45RA^-^ CCR7^-^) (**Figure 1D**). Among the aforementioned clusters, C18 (CD4^+^ Tcm) exhibited the expression of Ki67, whereas the remaining clusters did not demonstrate Ki67 expression. **Supplementary Table 2** provides a description of the 26 clusters representing T cells subpopulation.

It was observed that the count of C18 was notably lower in INRs, as depicted in **Supplementary Figure 2**. Taking into account the disparity in CD4^+^ T cell counts between the two groups, CD4^+^ Ki67^+^ T cells were manual gated (**Figure 1E**), and the proportion of CD4^+^ Ki67^+^ T cells was calculated as a percentage of CD4^+^ cells. As a result, it was observed that the proportion of CD4^+^ Ki67^+^ T cells was significantly higher in INRs (**Figure 1F**), and therefore, we defined the CD4^+^ Ki67^+^ T cells in the INR as over-proliferating cells. Differences in the frequencies of other cells subpopulation between the two groups are shown in **Supplementary Figure 2**. Specifically, the frequencies of C10 (CD4^+^ Tn) and C13 (CD4^+^ Tcm) were found to be significantly lower in INRs. No significant differences were observed in the frequencies of other subpopulations of CD4^+^ T cells between the IRs and INRs groups (data not presented). Although we intended to find whether there were differences of markers expression (HLA-DR, CD38, CD57, PD-1, PD-L1, CD107a, perforin) in Ki67^+^ CD4^+^ T cells between INRs and IRs, the results did not show significant differences.

### 3.3 Proliferating CD4^+^ T cells were in a status of poor quality in INRs

**Figure 2:**
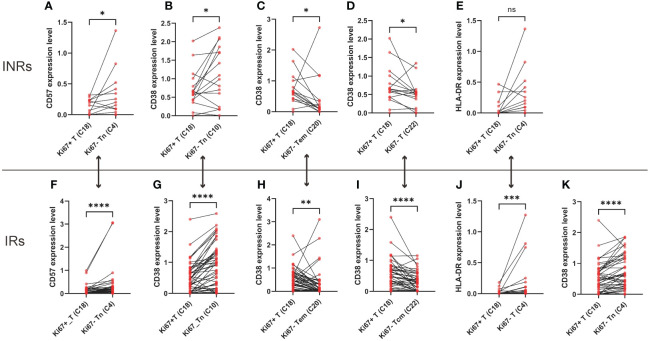
The over-proliferating CD4^+^ T cells exhibited higher levels of senescence, activation. **(A)** Difference of CD57 expression between Ki67^+^CD4^+^ T cells (C18) and Ki67^-^CD4^+^ naïve T cells (C4) in INRs. **(B)** Difference of CD38 expression between Ki67^+^CD4^+^ T cells (C18) and Ki67^-^CD4^+^ naïve T cells (C10) in INRs. **(C)** Difference of CD38 expression between Ki67^+^CD4^+^ T cells (C18) and Ki67^-^CD4^+^ effector memory T cells (C20) in INRs. **(D)** Difference of CD38 expression between Ki67^+^CD4^+^ T cells (C18) and Ki67^-^CD4^+^ central memory T cells (C22) in INRs. **(E)** Difference of HLA-DR expression between Ki67^+^CD4^+^ T cells (C18) and Ki67^-^CD4^+^ naïve T cells (C4) in INRs. **(F)** Difference of CD57 expression between Ki67^+^CD4^+^ T cells (C18) and Ki67^-^CD4^+^ naïve T cells (C4) in IRs. **(G)** Difference of CD38 expression between Ki67^+^CD4^+^ T cells (C18) and Ki67^-^CD4^+^ naïve T cells (C10) in IRs. **(H)** Difference of CD38 expression between Ki67^+^CD4^+^ T cells (C18) and Ki67^-^CD4^+^ effector memory T cells (C20) in INRs. **(I)** Difference of CD38 expression between Ki67^+^CD4^+^ T cells (C18) and Ki67^-^CD4^+^ central memory T cells (C22) in INRs. **(J)** Difference of HLA-DR expression between Ki67^+^CD4^+^ T cells (C18) and Ki67^-^CD4^+^ naïve T cells (C4) in INRs. **(K)** Difference of CD38 expression between Ki67^+^CD4^+^ T cells (C18) and Ki67^-^CD4^+^ naïve T cells (C4) in IRs. IRs: immunological responders; INRs: immunological non-responders. Data are shown as media and Interquartile range. Mann–Whitney U-test was used to test group difference. *p<0.05, **p<0.01, ***p<0.001, ****p<0.0001.

**Figure 3:**
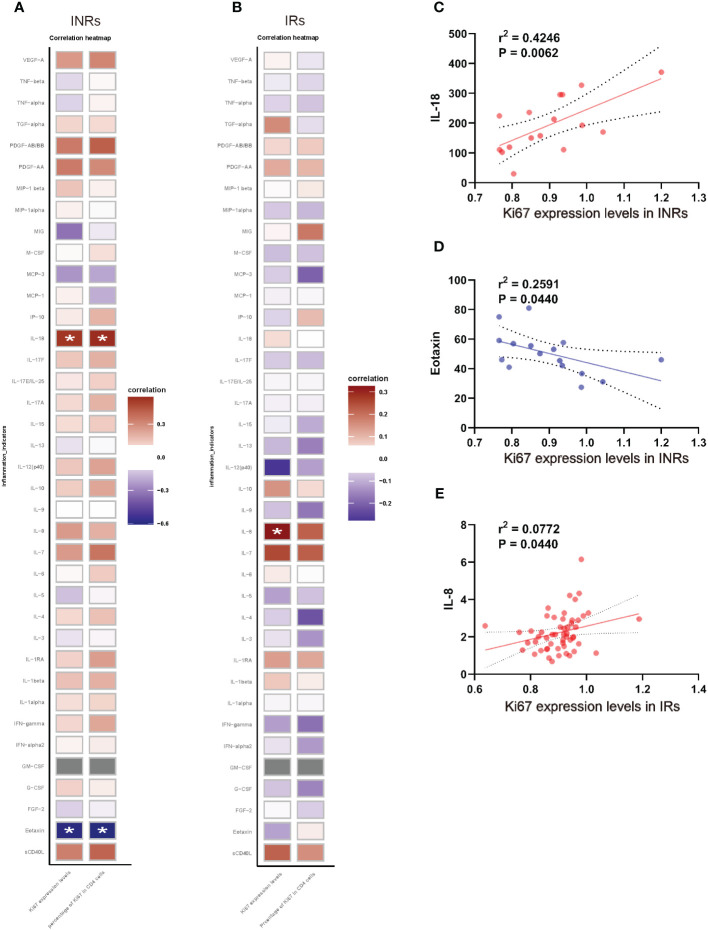
Expression level and proportion of Ki67^+^ CD4^+^ T cells correlated with chronic inflammation. **(A)** Correlation between the expression level, proportion of Ki67^+^ CD4^+^ T cell and cytokines/chemokines in INRs. **(B)** Correlation between the expression level, proportion of Ki67^+^ CD4^+^ T cell and cytokines/chemokines in IRs. **(C)** Correlation between IL-18 and Ki67 expression levels in INRs. **(D)** Correlation between eotaxin and Ki67 expression levels in INRs. **(E)** Correlation between IL-8 and Ki67 expression levels in INRs. IRs, immunological responders; INRs, immunological non-responders; IL, interleukin; IFN, interferon; TNF, tumor necrosis factor; G/M-CSF, granulocyte/macrophage colony-stimulating factor; VEGF, vascular endothelial growth factor; TGF, transforming growth factor; MCP, monocyte chemotactic protein; MIP, macrophage inflammatory protein Correlation analyzed by Spearman’s correlation. *p<0.05.

Given the absence of disparities in the expression of markers among the two groups, we proceeded to examine the expression of marker within CD4^+^ T cells that either expressed or did not express Ki67, respectively. We compared the markers’ expression of activation (CD38, HLA-DR), senescence (CD57), cytotoxicity (CD107A, perforin), and programmed apoptosis (PD-1, PD-L1) between clusters with Ki67^+^ CD4^+^ T cells (C18) and Ki67^-^CD4^+^ T cells in INRs (**Figures 3A–D**). When comparing to the Ki67^-^ CD4^+^ Tn cells (C4, C10), Ki67^+^ CD4^+^ T cells exhibited decreased expression of CD57 (**Figure 2A**) and CD38 (**Figure 2B**), whereas this difference did not appear between Ki67^+^ CD4^+^ T cells and Ki67^-^ CD4^+^ Tcm cells (C13, C22, C25). Furthermore, Ki67^-^ CD4^+^ Tcm cells and Tem cells showed the opposite difference compared with Ki67^+^ CD4^+^ T cells, C20 and C22 exhibited decreased levels of CD38 (**Figures 2C, D**). C18 and C4 did not show difference of HLA-DR expression in INRs (**Figure 2E**). It was observed that INRs revealed a higher susceptibility to senescence in Ki67^-^ cells when CD4^+^ T cells were in the immature Tn cells stage. However, this difference ceased to exist when CD4^+^ Tn cells differentiated into immune-competent Tcm cells or Tem cells. These findings suggested that CD4^+^ T cells, which experience over-proliferation in INRs, also display an elevated degree of activation. Such proliferating cells, although more numerous in INRs, may have higher levels of senescence and activation and thus be in a state of poor quality.

### 3.4 Certain Tcm cells in IRs exhibited low-quality

We further compared the markers’ expression of activation (CD38, HLA-DR), senescence (CD57), cytotoxicity (CD107A, perforin), and programmed apoptosis (PD-1) between clusters with Ki67^+^CD4^+^ T cells and Ki67^-^CD4^+^ T cells in IRs (**Figures 3F–K**). Similar to INRs, IRs also demonstrated elevated levels of CD38, HLA-DR and CD57 in CD4^+^ Tn cells (**Figures 2F, G, J, K**). In contrast to INRs, in IRs, there was a lower level of CD38 of Ki67^+^CD4^+^ Tcm cells (C18), compared to Ki67^-^ CD4^+^ Tcm cells (C20) (**Figure 2H**). This finding demonstrated a similar alignment with our hypothesis. The observation of poor quality in Ki67^+^ T cells within the INRs was also noted in IRs (compared with C13, C22, C25). Notably, this distinction was not observed in all subpopulations in IRs, opposite trend was even shown in some central memory Tcm cells (C20). Within IRs, Ki67^+^ T cells exhibited lower CD38 levels compared to C20, whereas in INRs, CD38 expression levels were conversely higher. This further supports the notion that heightened activation levels, alternatively, worse quality of Ki67^+^ T cells in INRs.

### 3.5 Over-proliferating CD4^+^ T cells correlated with the chronic inflammation in INRs

Given that HIV infection leads to chronic immune activation and increased release of inflammatory cytokines, particularly in INRs (Yang et al., 2020), we further analyzed the association between the expression of Ki67 and cytokines/chemokines in both INRs and IRs, respectively (**Figure 3**). The results revealed that, among INRs, both the expression levels of Ki67^+^ and the percentage of Ki67 in CD4^+^ T cells exhibited positive correlations with IL-18 and negative correlations with eotaxin (**Figures 3A, C, D**). Additionally, expression levels of Ki67^+^ were positively correlated with IL-8 in IRs (**Figures 3B, E**). These results suggested that elevated levels of chronic inflammation were associated with abnormal proliferation of CD4^+^ T cells.

## 4 Discussion

This article puts forward the hypothesis of “pathological proliferation” in INRs: excessive proliferation of CD4^+^ T cells in INRs may be accompanied by aberrant activation, and loss of immune function. Eventually, the CD4^+^ T cell counts and function of INRs cannot be fully restored. To test the hypothesis of “pathological proliferation,” we measured the number and function of CD4^+^ T cells in the peripheral blood of INRs and IRs, as well as the levels of cytokines and chemokines Firstly, INRs demonstrated a greater proportion of Ki67^+^ CD4^+^ T cells subpopulation, which we have designated as over-proliferating CD4^+^ T cells. Secondly, within cells that have differentiated into CD4^+^ Tcm cells, those over-proliferating Ki67^+^ CD4^+^ T cells were in status of poor quality. Additionally, it was observed that certain Tcm cells in IRs exhibited the opposite difference to INRs. Furthermore, there were significant correlations between the quantities and proportions of over-proliferating CD4^+^ T cells and the presence of chronic inflammation.

The proportions of over-proliferating CD4^+^ T cell subsets was found to be increased in INRs, along with a reduction in total CD4 T cell counts. The present study observed that INRs had significantly lower initial/recent CD4 T cell counts compared to IRs, somehow a significantly higher proportion of CD4^+^ T cells in their subset of expressing Ki67. Ki67 represented the ability of cell proliferation (Scholzen and Gerdes, 2000), thus we classified CD4^+^ T cells with significantly higher Ki67 expression in INRs as over-proliferating cells. Previous studies have suggested that homeostatic T-cell proliferation can occur in conditions of lymphopenia (Bell et al., 1987; Rocha et al., 1989), encompassing both naive and memory T cells (Kieper and Jameson, 1999; Ernst et al., 1999). In the context of HIV infection, the depletion of T cells is linked to the homeostatic response of T cells. Specifically, there is evidence of an inverse correlation between the count of CD4^+^ T cells and the quantity of CD4^+^ cycling T cells (Anthony et al., 2003; Sousa et al., 2002). Individuals who were unable to restore their CD4^+^ T cell counts exhibited higher rates of homeostatic proliferation compared to those who responded to treatment and successfully reconstructed their CD4^+^ T cells pool (Marchetti et al., 2006). A previous study has demonstrated that inadequate restoration of T cell counts and function in INRs can result in the proliferation of active T cells in order to achieve “homeostasis” (Saidakova et al., 2018). In our study, a comparable phenomenon was noted, wherein INRs exhibited a greater depletion of CD4^+^ T cells; however, the proportion of proliferating cells within CD4^+^ T cells was found to be higher in INRs when compared to IRs.

Over-proliferating but poor-quality cells caused the insufficient restoration of CD4 levels in INRs. In individuals infected with HIV, CD4^+^ T cells exhibit both heightened activation and senescence alongside their excessive proliferation, this aberrant condition is particularly pronounced in INRs. Our investigation of INRs revealed that differentiated mature Ki67^+^ CD4^+^ Tcm cells, also display elevated expression of CD38 and CD57, indicative of a greater degree of activation and senescence in the over-proliferating CD4^+^ T cells. Besides, similar features were observed in IRs, but certain Tcm cells in IRs also showed the opposite. Senescence, a condition marked by compromised T cell function (Palmer et al., 2005), is prevalent in HIV infection (Papagno et al., 2004). Previous research has indicated that excessive T-cell proliferation can also contribute to cellular senescence (Effros and Pawelec, 1997). HIV infection induces continuous and active proliferation, leading to the accumulation of T lymphocytes that are characterized as “senescent” (Papagno et al., 2004; Nakanjako et al., 2011). This persistent immune activation is linked to inadequate immune restoration in individuals with HIV. Even with antiretroviral therapy (ART), T cells activation remains insufficient for the recovery of CD4^+^ T cells (Marchetti et al., 2006; Goicoechea et al., 2006; Ancuta et al., 2008). Consequently, our findings propose a phenomenon wherein over-proliferated CD4^+^ T cells in individuals with incomplete immune reconstitution also display heightened levels of persistent activation and senescence. Based on the above findings, our study revealed that the over-proliferation of CD4^+^ T cells in INRs were accompanied by heightened levels of senescence and activation. Consequently, these over-proliferating but poor-quality cells resulted in the restoration of CD4 levels in INRs failing to reach the same magnitude as observed in IRs. This aligns with our hypothesis of “pathological proliferation”. Regrettably, our study did not yield significant disparities in the function of Ki67^+^ CD4^+^ T cells between INRs and IRs. Consequently, further investigations are imperative to comprehensively characterize the CD4^+^ T cells undergoing “pathological proliferation”.

The expression of Ki67 in CD4^+^ T cells exhibited a significant association with chronic inflammation in INRs. Our investigation revealed that in INRs, the expression levels of Ki67 in CD4^+^ T cells and the proportions of Ki67^+^ CD4^+^ T cells demonstrated positive correlation with IL-18 levels and a negative correlation with eotaxin levels. In IRs, the expression of Ki67 in CD4^+^ T cells exhibited a positive correlation with IL-8 levels. Previous research has indicated that cytokines derived from antigen-presenting cells (APCs) can directly enhance T cell responses, primarily following co-stimulation from accessory molecule co-receptors (Joseph et al., 1998). Additionally, pro-inflammatory cytokines such as IL-2, IL-6, and TNF have been shown to promote T cell proliferation (Joseph et al., 1998). Persistent immune activation and inflammation are characteristic features of chronic HIV infection and serve as reliable predictors of disease progression, irrespective of plasma HIV load or peripheral CD4^+^ T cell counts (Pandrea and Apetrei, 2010). Our study further revealed that CD4^+^ T cells exhibiting over-proliferation in INRs also exhibited abnormal immune activation, the expression of Ki67 in these cells exhibited a positive correlation with the pro-inflammatory cytokine IL-18, while displaying a negative correlation with eotaxin. This may be a feature of pathologically proliferating CD4^+^ T cells in INRs, yet the underlying mechanism remains unclear, necessitating further investigation. Simultaneously, this distinctive attribute holds promise in establishing a theoretical framework for prognosticating the likelihood of inadequate immune reconstitution in clinical settings.

To the best of our understanding, this is the first proposal of the hypothesis of “pathological proliferation” in the context of HIV infection. This hypothesis specifically directed attention towards the “pathologically proliferating” CD4^+^ T cells, that were in a state of low quality despite over-proliferation. Such proliferating cells were unable to fully function, resulting in impaired recovery of CD4 in INRs. By combining the features of HIV infection and immunity, this hypothesis not only aligns with the current trajectory of AIDS research in the post-ART era and the imperatives of national health, but also introduces novel scientific perspectives on the immune mechanism and immunotherapy of infectious diseases.

The study’s limitations and potential avenues for future research in the field warrant consideration. Primarily, this study concentrated on the pathological proliferation phenomenon within T cells, leaving the inquiry into the existence of the same phenomenon within monocytes for subsequent investigations. Additionally, the demographic characteristics of the two groups did not exhibit perfect congruence. Considering the limited number of participants in this study and the substantial disparity in subject numbers between the INR and IR groups, it is imperative to conduct future research with a larger sample size, to enhance the reliability and applicability of the findings in this study. Furthermore, in this study, some inflammatory factors were detected and their correlation with “pathologically proliferating” cells was analyzed, but the cytokines detected were not comprehensive, and the detection of related cytokines (e.g., IL-2) needs to be further improved in future studies, so that we could have a more comprehensive understanding of the association between “pathological proliferation” and inflammation. In future investigations, it is crucial to identify prospective targets for the restoration of CD4^+^ T cell levels in HIV-infected individuals experiencing poor immune reconstitution, employing the characterization of pathologically proliferating cells. Additionally, it is imperative to examine the efficacy of diverse therapeutic strategies in facilitating the recovery of CD4 cell counts.

## 5 Conclusion

This study presented and substantiated the hypothesis of CD4^+^ T cells “pathological proliferation” in INRs. Specifically, our findings indicate an over-proliferation of CD4^+^ T cells in INRs. Over-proliferating CD4^+^ T cells were accompanied by aberrant activation and senescence, resulting in a state of diminished function. This lead to the incomplete restoration of CD4 levels. Therefore, further characterization of CD4^+^ T cells pathological proliferation and reduction of this heterogeneity have emerged as a major priority for promoting immune reconstitution in PLWH.

## Data availability statement

The original contributions presented in the study are included in the article/**Supplementary Material**. Further inquiries can be directed to the corresponding authors.

## Ethics statement

The studies involving humans were approved by the Ethics Committee of Beijing Youan Hospital, Capital Medical University (2023/057). The studies were conducted in accordance with the local legislation and institutional requirements. The participants provided their written informed consent to participate in this study.

## Author contributions

YZ: Writing – original draft, Visualization, Supervision, Software, Resources, Project administration, Methodology, Investigation, Funding acquisition, Formal analysis, Data curation, Conceptualization. JJ: Visualization, Validation, Software, Methodology, Investigation, Formal analysis, Data curation, Conceptualization, Writing – original draft. KX: Visualization, Validation, Software, Investigation, Formal analysis, Data curation, Writing – review & editing. MC: Supervision, Resources, Project administration, Methodology, Investigation, Formal analysis, Writing – review & editing. RW: Visualization, Software, Methodology, Investigation, Data curation, Writing – review & editing. XZ: Visualization, Software, Methodology, Data curation, Writing – review & editing. XC: Visualization, Software, Methodology, Data curation, Writing – review & editing. YLZ: Visualization, Supervision, Resources, Methodology, Writing – review & editing. HW: Writing – review & editing, Supervision, Resources, Methodology, Funding acquisition. WW: Writing – review & editing, Writing – original draft, Validation, Supervision, Software, Project administration, Methodology, Investigation, Formal analysis, Data curation, Conceptualization. ZL: Writing – review & editing, Writing – original draft, Visualization, Validation, Supervision, Software, Project administration, Methodology, Investigation, Formal analysis, Data curation, Conceptualization. TZ: Writing – review & editing, Writing – original draft, Validation, Supervision, Software, Resources, Project administration, Methodology, Investigation, Funding acquisition, Data curation, Conceptualization.
