# Supplementary material for: Pathological proliferation: a potential mechanism for poor CD4+ T cell recovery in people living with HIV
Source: Front Cell Infect Microbiol. 2024 Mar 27;14:1344778. doi: 10.3389/fcimb.2024.1344778 (PMC11004319; doi:10.3389/fcimb.2024.1344778)
Supplement: Supplementary file 1 [file DataSheet_1.docx]

Supplementary Material

## Supplementary Figures

**Supplementary Figure 1.** Gating strategy for data cleaning to identify CD3+ T cells from PBMC. (A) AEQ3 beads andEQ4 beads were utilized to recognize cell events from all events. (B-C) Single living cells were recognized by gating the cell events through 193Ir vs. Event_length and 195Pt. (D) CD45^+^ cells were gated from the single living cells. (E) (D) CD3^+^ cells were gated from the CD45^+^ cells

## Supplementary Tables

**Supplementary Table 1** Antibodies and isotopes used for mass cytometry

|  | Antibodies Name | Isotopes Name | Clone | Company | Size (Tests) |
| --- | --- | --- | --- | --- | --- |
| 1 | Anti-Human CD16 | 209Bi | 3GB | Fluidigm, South San Francisco, United States | 100 |
| 2 | Anti-Human CD57 | 142Nd | HCD57 | Fluidigm, South San Francisco, United States | 100 |
| 3 | Anti-Human KI67 | 161Dy | B56 | Fluidigm, South San Francisco, United States | 100 |
| 4 | Anti-Human FOXP3 | 162Dy | PCH101 | Fluidigm, South San Francisco, United States | 50 |
| 5 | Anti-Human CCR7 | 167Er | G403H7 | Fluidigm, South San Francisco, United States | 50 |
| 6 | Anti-Human CD127 | 168Er | A019D5 | Fluidigm, South San Francisco, United States | 100 |
| 7 | Anti-Human HLA-DR | 170Er | L243 | Fluidigm, South San Francisco, United States | 100 |
| 8 | Anti-Human CD107A | 151Eu | H4A3 | Fluidigm, South San Francisco, United States | 100 |
| 9 | Anti-Human CCR2 | 153Eu | K036C2 | Fluidigm, South San Francisco, United States | 100 |
| 10 | Anti-Human CD163 | 154Sm | GHI/61 | Fluidigm, South San Francisco, United States | 100 |
| 11 | Anti-Human PD-1 | 155Gd | EH12.2H7 | Fluidigm, South San Francisco, United States | 100 |
| 12 | Anti-Human PD-L1 | 156Gd | 29E.2A3 | Fluidigm, South San Francisco, United States | 100 |
| 13 | Anti-Human PERFORIN | 175Lu | B-D48 | Fluidigm, South San Francisco, United States | 100 |
| 14 | Anti-Human CD45RA | 143Nd | Hl100 | Fluidigm, South San Francisco, United States | 100 |
| 15 | Anti-Human CD4 | 145Nd | RPA-T4 | Fluidigm, South San Francisco, United States | 100 |
| 16 | Anti-Human CD8 | 146Nd | RPA-T8 | Fluidigm, South San Francisco, United States | 100 |
| 17 | Anti-Human CD3 | 141Pr | UCHT1 | Fluidigm, South San Francisco, United States | 100 |
| 18 | Anti-Human CD31 | 144Nd | WM59 | Fluidigm, South San Francisco, United States | 100 |
| 19 | Anti-Human CD14 | 148Nd | RM052 | Fluidigm, South San Francisco, United States | 100 |
| 20 | Anti-Human CD25 | 149Sm | 2A3 | Fluidigm, South San Francisco, United States | 100 |
| 21 | Anti-Human CD86 | 150Nd | IT2.2 | Fluidigm, South San Francisco, United States | 100 |
| 22 | Anti-Human CD45 | 89Y | HI30 | Fluidigm, South San Francisco, United States | 100 |
| 23 | Anti-Human CD38 | 172Yb | HIT2 | Fluidigm, South San Francisco, United States | 100 |

**Supplementary Table 2** CD3+ T cell and monocyte clusters of all participants(1)

|  | Subpopulation | Clusters | Markers |
| --- | --- | --- | --- |
| CD4^+^ T cells | Naïve T cells | C4, C10 | CD3^+^ CD4^+^ CD8^-^ CD45RA^+^ CCR7^+^ |
|  | Central memory T cells | C13, C18, C22, C25 | CD3^+^ CD4^+^ CD8^-^ CD45RA^-^ CCR7^+^ |
|  | Effector memory T cells | C20 | CD3^+^ CD4^+^ CD8^-^ CD45RA^-^ CCR7^-^ |
| CD8^+^ T cells | Naïve T cells | C3, C6, C7, C8, C11, C21 | CD3^+^ CD4^-^ CD8^+^ CD45RA^+^ CCR7^+^ |
|  | CD45RA^+^ effector memory T cells | C9, C12, C15, C16, C17, C19, C23 | CD3^+^ CD4^-^ CD8^+^ CD45RA^+^ CCR7^-^ |
|  | Central memory T cells | C5 | CD3^+^ CD4^-^ CD8^+^ CD45RA^-^ CCR7^+^ |
|  | Effector memory T cells | C26 | CD3^+^ CD4^-^ CD8^+^ CD45RA^-^ CCR7^-^ |
| CD4^+^ CD8^+^ T cells |  | C14 | CD3^+^ CD4^+^ CD8^+^ |
| CD4^-^ CD8^-^ T cells |  | C1, C2, C24 | CD3^+^ CD4^-^ CD8^-^ |

**Reference**

1. Cohen EEW, Pishvaian MJ, Shepard DR, Wang D, Weiss J, Johnson ML, et al. A Phase Ib Study of Utomilumab (Pf-05082566) in Combination with Mogamulizumab in Patients with Advanced Solid Tumors. *Journal for immunotherapy of cancer* (2019) 7(1):342. Epub 2019/12/06. doi: 10.1186/s40425-019-0815-6.
