# Supplementary figures and images for: Pathological proliferation: a potential mechanism for poor CD4+ T cell recovery in people living with HIV
Source: Front Cell Infect Microbiol. 2024 Mar 27;14:1344778. doi: 10.3389/fcimb.2024.1344778 (PMC11004319; doi:10.3389/fcimb.2024.1344778)

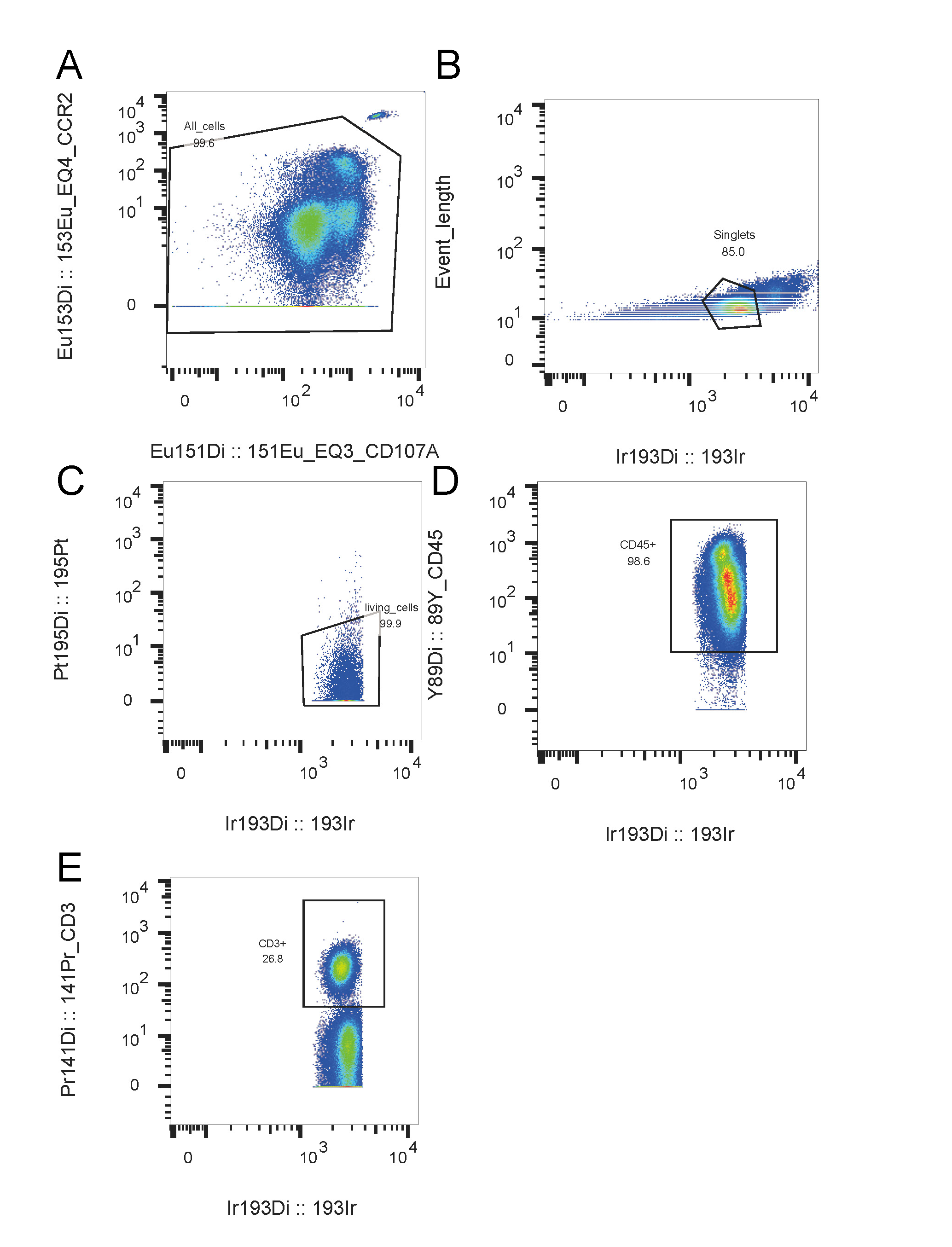

Supplement: Supplementary file 2 [file Image_1.tif]

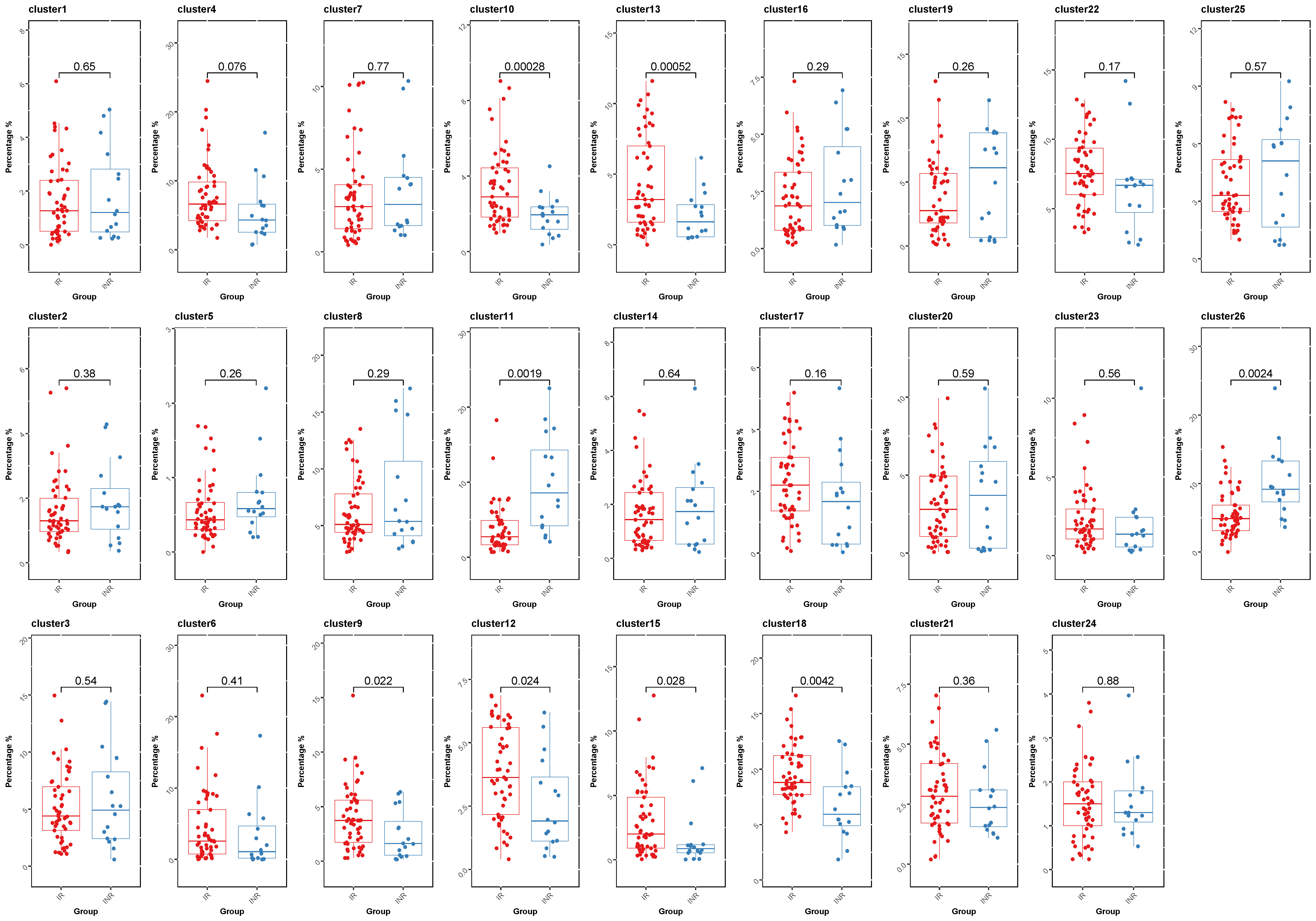

Supplement: Supplementary file 3 [file Image_2.jpeg]
